# Zinc transporters belonging to the Cation Diffusion Facilitator (CDF) family have complementary roles in transporting zinc out of the cytosol

**DOI:** 10.1371/journal.pgen.1007262

**Published:** 2018-03-12

**Authors:** Sangyong Choi, Ya-Mei Hu, Mark E. Corkins, Amy E. Palmer, Amanda J. Bird

**Affiliations:** 1 Department of Human Nutrition, The Ohio State University, Columbus, Ohio, United States of America; 2 Department of Molecular Genetics, The Ohio State University, Columbus, Ohio, United States of America; 3 Department of Chemistry and Biochemistry and BioFrontiers Institute, University of Colorado Boulder, Boulder, Colorado, United States of America; 4 Center for RNA Biology, The Ohio State University, Columbus, Ohio, United States of America; Washington University, UNITED STATES

## Abstract

Zinc is an essential trace element that is required for the function of a large number of proteins. As these zinc-binding proteins are found within the cytosol and organelles, all eukaryotes require mechanisms to ensure that zinc is delivered to organelles, even under conditions of zinc deficiency. Although many zinc transporters belonging to the Cation Diffusion Facilitator (CDF) families have well characterized roles in transporting zinc into the lumens of intracellular compartments, relatively little is known about the mechanisms that maintain organelle zinc homeostasis. The fission yeast *Schizosaccharomyces pombe* is a useful model system to study organelle zinc homeostasis as it expresses three CDF family members that transport zinc out of the cytosol into intracellular compartments: Zhf1, Cis4, and Zrg17. Zhf1 transports zinc into the endoplasmic reticulum, and Cis4 and Zrg17 form a heterodimeric complex that transports zinc into the cis-Golgi. Here we have used the high and low affinity ZapCY zinc-responsive FRET sensors to examine cytosolic zinc levels in yeast mutants that lack each of these CDF proteins. We find that deletion of *cis4* or *zrg17* leads to higher levels of zinc accumulating in the cytosol under conditions of zinc deficiency, whereas deletion of *zhf1* results in zinc accumulating in the cytosol when zinc is not limiting. We also show that the expression of *cis4*, *zrg17*, and *zhf1* is independent of cellular zinc status. Taken together our results suggest that the Cis4/Zrg17 complex is necessary for zinc transport out of the cytosol under conditions of zinc-deficiency, while Zhf1 plays the dominant role in removing zinc from the cytosol when labile zinc is present. We propose that the properties and/or activities of individual CDF family members are fine-tuned to enable cells to control the flux of zinc out of the cytosol over a broad range of environmental zinc stress.

## Author summary

All organisms require homeostasis mechanisms to maintain sufficient levels of zinc for normal cell metabolism and to avoid toxicity. As zinc-binding proteins are located in the cytosol and within intracellular compartments, all cells have to balance intracellular zinc ion distribution so that there are sufficient, but non toxic levels of zinc in the cytosol as well as organelles. Although much is known about the mechanisms that control cytosolic zinc levels, relatively little is known about the mechanisms that maintain organelle zinc homeostasis. As proteins belonging to the CDF family transport zinc into organelles, here we used a fission yeast model system to determine if the expression or function of zinc transporters belonging to this family was regulated by zinc. We find that two CDF family members, Cis4 and Zrg17, facilitate the transport of zinc out of the cytosol of zinc-deficient cells, whereas the CDF family member Zhf1 preferentially transports zinc out of the cytosol when zinc is not limiting. As the expression of the genes encoding these transport proteins is not regulated by zinc, the results suggest that different CDF family members have complementary roles in transporting zinc out of the cytosol that are independent of changes in transcription. These results provide new insights into the regulatory mechanisms that control cytosolic and organelle zinc homeostasis.

## Introduction

Zinc is an essential trace metal that is required for the structure and activity of a large number of proteins. In eukaryotes these proteins include transcription factors containing structural domains stabilized by zinc ions, such as the C_2_H_2_-type and C_4_-type zinc fingers [1]. Zinc is also a cofactor for many enzymes that are located in the cytosol (e.g. alcohol dehydrogenase 1), and in subcellular compartments such as the nucleus (e.g. RNA polymerases), mitochondria (e.g. cytochrome c oxidase), and endoplasmic reticulum (e.g. calreticulin) [2–4]. Due to the essential nature of some of these proteins, all organisms are challenged with obtaining sufficient levels of zinc for incorporation into newly synthesized proteins. A further complicating factor is that excessive levels of zinc are toxic to cells. As a consequence, zinc acquisition, compartmentalization, storage, and efflux need to be tightly regulated to maintain zinc at a level that is sufficient, but not toxic to cell metabolism.

In many organisms zinc-responsive transcription factors maintain zinc homeostasis by controlling the expression of genes that are required for the transport of zinc into and out of the cytosol. In eukaryotes these zinc transport proteins commonly belong to either the Zrt- Irt- like protein family (ZIP) or CDF family. Members of the ZIP family typically facilitate zinc uptake or the release of zinc from intracellular stores, whereas the CDF family members usually transport zinc into the lumens of intracellular compartments or out of a cell [5]. As zinc transport by a ZIP family member typically results in an increase in cytosol zinc levels, the expression of genes encoding ZIP family members is often up-regulated when zinc is limiting [6]. As an example, in *Saccharomyces cerevisiae* the transcriptional activator Zap1 controls the expression of genes encoding ZIP family members required for zinc uptake (Zrt1 and Zrt2) and release of zinc from the vacuolar stores (Zrt3) [7]. As Zap1 is active in zinc-limited cells and is inactive when zinc is in excess, the expression of *ZRT1-3* increases when cells need zinc. Importantly, as zinc transport into the cytosol by the ZIP proteins inactivates Zap1, a negative feedback loop is created that prevents zinc from reaching toxic levels.

Negative feedback circuits also control the expression of CDF family members. In humans, the metal-responsive transcription factor 1 (MTF-1) regulates the expression of ZnT1, an essential CDF family member that is required for zinc efflux from cells [8]. MTF-1 is activated by excess zinc in the cytosol, which in turn transcriptionally induces *ZnT1* expression when zinc is high. Similarly, when dietary zinc levels are high in the nematode *Caenorhabditis elegans*, the high zinc-responsive factor 1 (HIZR-1) induces the expression of CDF family members required for the excretion of zinc from intestinal cells (*ttm-1b)* and storage of zinc in intestinal gut granules (*cdf-2*) [9]. As the end result of these transcriptional changes is a reduction in cytosolic zinc levels, thereby inactivating MTF-1 and HIZR-1, a negative feedback loop is created that prevents the cytosol from being depleted of zinc.

Although a number of genes encoding transporters required for zinc uptake, storage, and efflux are subject to negative feedback control, the expression of some CDF family members increases in zinc-limited cells. For example, Zrg17 and Msc2 are two CDF family members from *S*. *cerevisiae* that form a heterodimeric complex that transports zinc into the endoplasmic reticulum [10]. Although this complex transports zinc out of the cytosol, *ZRG17* is a Zap1-target gene that is expressed at higher levels in zinc-deficient cells [11]. While this regulation at first seems counterintuitive, as it would further deplete zinc from the cytosol, the induction of *ZRG17* by Zap1 is critical for preventing the unfolding of proteins in the endoplasmic reticulum under this condition [11]. As zinc transport by the Zrg17/Msc2 complex would also further increase Zap1 activity, the zinc-dependent regulation of *ZRG17* presumably results in a positive feedback circuit to supply zinc to compartmentalized proteins when the cytosol is limited for zinc.

The regulation of *ZRG17* by Zap1 illustrates a mechanism of how zinc can be supplied to an intracellular compartment in a zinc-limited environment. As few other studies have examined the regulatory circuits that maintain zinc levels in organelles during periods of zinc starvation, the goal of this work was to determine if related mechanisms were present in the distantly related yeast *S*. *pombe*. We chose to use *S*. *pombe* because multiple aspects of zinc homeostasis differ between fission and budding yeast. These differences include the transcription factor used to control zinc homeostasis (Loz1 vs. Zap1), the primary site for the storage of excess zinc (endoplasmic reticulum vs. vacuole), and the presence of metallothioneins that preferentially bind divalent metal ions such as zinc (Zym1 from *S*. *pombe*) or monovalent ions such as copper (Cup1 from *S*. *cerevisiae*) [12–16]. Another difference between fission and budding yeasts is the subcellular localization of zinc transporters within the secretory pathway. In *S*. *cerevisiae* two CDF family members, Zrc1 and Cot1, transport zinc into the vacuole [17]. In *S*. *pombe*, the homolog of Zrc1, named Zhf1, transports zinc into the endoplasmic reticulum, and the homologs of Msc2 and Zrg17 (named Cis4 and Zrg17 respectively) form a complex that localizes to the cis-Golgi [18, 19]. The biological significance of these differences in subcellular localization of the CDF family members between budding and fission yeasts is currently unclear.

To gain a better understanding of the mechanisms that control the supply of zinc to organelles, we used multiple genetic approaches to determine the extent to which three CDF family members from *S*. *pombe* (Zhf1, Zrg17, and Cis4) facilitate zinc transport out of the cytosol under conditions of zinc deficiency and zinc excess. We found that deletion of *zhf1* results in a strong growth defect when zinc is in excess and that deletion of *zrg17* or *cis4* leads to a mild growth defect in the presence of the zinc chelator EDTA. These latter results suggest that the Cis4/Zrg17 complex may play an important role under zinc deficiency conditions. To further investigate whether transport via Cis4 and Zrg17 is affected by cellular zinc status we developed methods to monitor changes in cytosolic zinc availability in fission yeast. These analyses revealed that that *cis4*Δ and *zrg17*Δ cells accumulate higher levels of zinc in the cytosol under conditions of zinc deficiency, while *zhf1*Δ cells accumulate higher levels of zinc in the cytosol when zinc is not limiting. We also show that the transcription of *zhf1*, *cis4*, and *zrg17* genes is not dependent upon zinc. These results reveal that different CDF family members have complementary roles in transporting zinc out of the cytosol. They also suggest that either the activities or the properties of different CDF family members are fine-tuned to transport zinc out of the cytosol under different environmental zinc stresses.

## Results

### Zinc-dependent phenotypes of zinc homeostasis mutants

**Fig 1:**
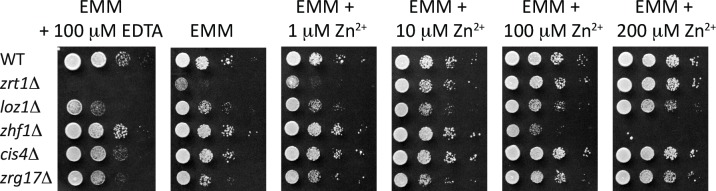
Growth phenotypes of yeast mutants with impaired zinc homeostasis. 10-fold serial dilutions of the indicated strains were plated onto Edinburgh Minimal Medium (EMM) supplemented with 100 μM EDTA or the indicated level of Zn^2+^. Plates were incubated for 3–5 days at 31°C before photography.

Three members of the CDF family transport zinc into the secretory pathway in fission yeast: Zhf1, Cis4, and Zrg17 [13, 15, 18, 19]. Although previous studies have shown that Zhf1 is required for growth in the presence of high zinc, relatively little was known about the roles of Cis4 and Zrg17 in zinc homeostasis. To determine if Cis4 and Zrg17 were necessary for growth under low or high zinc conditions, serial dilutions of *cis4*Δ and *zrg17*Δ cells were plated onto zinc-limiting (EMM + 100 μM EDTA) or zinc-replete medium (EMM + 0–200 μM zinc) (Fig 1). Cells lacking the Zrt1 or Zhf1, which are required for survival during zinc deficiency or zinc toxicity respectively [18], were also plated as controls. In the presence of 100 μM EDTA, *cis4*Δ cells exhibited a slight growth defect relative to the wild-type. z*rg17*Δ cells had a modest growth defect under all conditions, but grew more slowly in the presence of EDTA relative to *cis4*Δ. As Cis4 and Zrg17 form a heteromeric complex, these results are consistent with Cis4 and Zrg17 playing an important role in supplying zinc to the secretory pathway when zinc is limiting. The slower growth of *zrg17*Δ relative to *cis4*Δ also suggests that Zrg17 may have additional functions that are independent of Cis4 and zinc.

### The ZapCY1 and ZapCY2 zinc-responsive FRET sensors detect dynamic changes in the labile intracellular zinc pool

**Fig 2:**
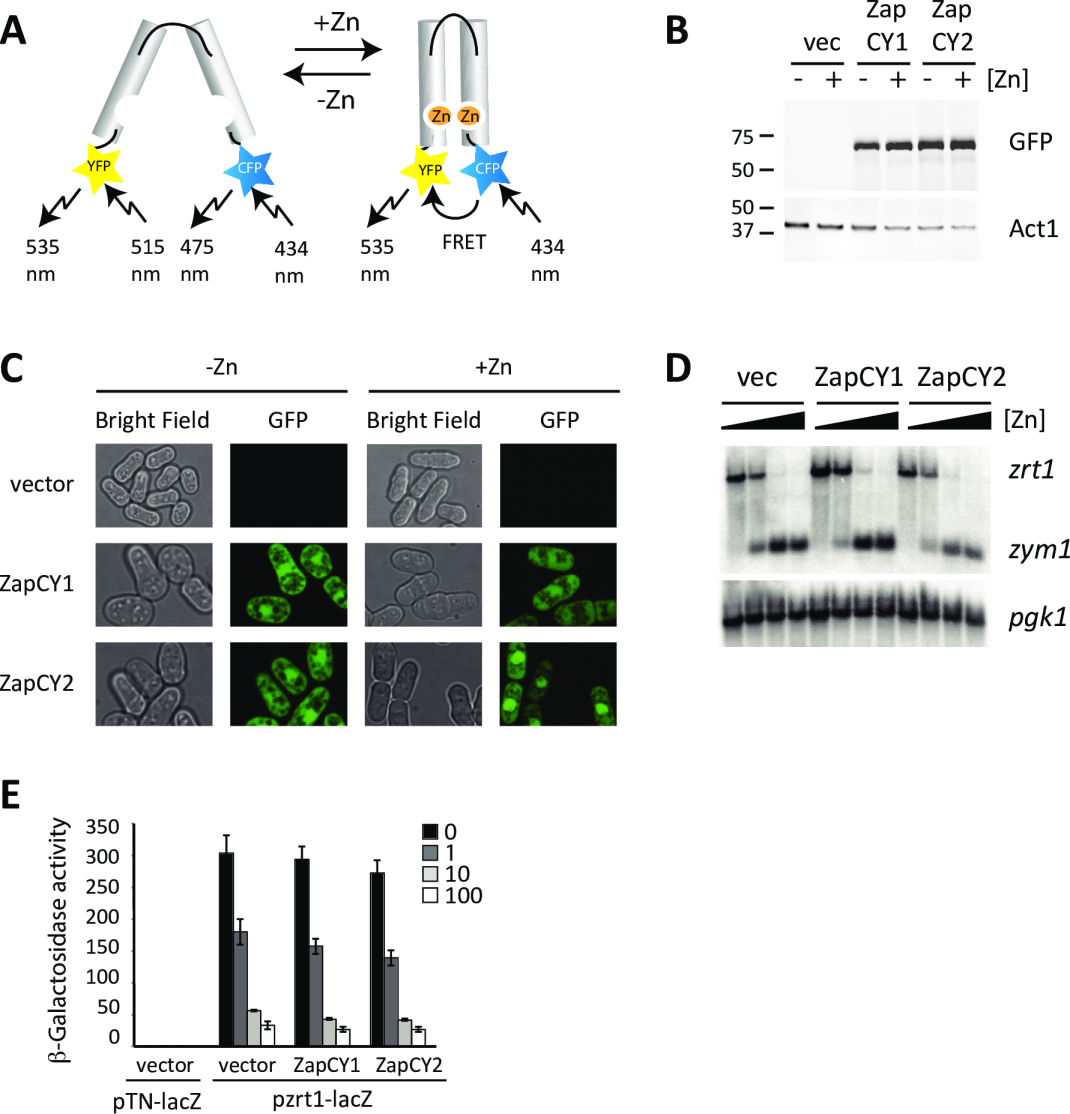
Expression of the ZapCY1 and ZapCY2 FRET reporters in fission yeast. **(A)** Schematic diagram of the ZapCY1 FRET sensor. Zinc fingers are shown as gray cylinders, and YFP and CFP are shown as yellow and blue colored stars. **(B)** Wild-type cells expressing ZapCY1, ZapCY2, or the empty vector were grown overnight in ZL-EMM without (-Zn) or with a 100 μM Zn^2+^ supplement (+Zn). Cells were collected and crude protein extracts prepped for immunoblot analysis. Immunoblots were probed with antibodies raised against GFP and the loading control Act1 (Actin). A protein ladder with sizes in kDa is shown on the left **(C)** Wild-type cells expressing the indicated reporters or the vector were grown in ZL-EMM (-Zn) or ZL-EMM + 100 μM Zn^2+^ (+Zn) and were analyzed by fluorescence microscopy (GFP). Bright field images are also shown. **(D)** Wild-type cells expressing ZapCY1, ZapCY2, or an empty vector (vec) were grown in ZL-EMM supplemented with 0, 1, 10, or 100 μM Zn^2+^ and total RNA purified for RNA blot analysis. RNA blots were probed for the zinc-regulated *zrt1* and *zym1* transcripts, and loading control *pgk1*. **(E)** β-galactosidase activity was measured in wild-type cells co-expressing a *zrt1-lacZ* reporter with the empty vector, ZapCY1, or ZapCY2. Cells were grown as described in panel D. Each bar shows the average value from three independent experiments and error bars show the standard deviations.

To determine if Cis4 and Zrg17 were required for zinc transport out of the cytosol of zinc-limited cells, we developed constructs to express the genetically encoded ZapCY1 and ZapCY2 zinc-responsive FRET sensors in the cytosol of fission yeast. The ZapCY1/2 sensors have been widely used to monitor dynamic changes in intracellular zinc levels [20]. Both sensors contain the regulatory zinc fingers 1 and 2 from the transcription factor Zap1, flanked by citrine YFP, hereafter referred to as YFP, and eCFP (Fig 2A). In zinc-limited environments, the Zap1 zinc finger domain 1 and 2 are largely unstructured. However, in the presence of zinc, the zinc finger domains fold together to form a single structural unit [21]. As this closed conformation brings together YFP and eCFP increasing FRET, the FRET signal in cells expressing ZapCY1 and ZapCY2 is directly coupled to intracellular zinc availability. Previous studies have shown that ZapCY1 binds zinc in vitro with an apparent dissociation constant of ~ 2.5 pM, while ZapCY2 contains substitutions within the zinc finger domains, which result in it binding zinc with an ~ 300 fold lower affinity [20].

To determine if the ZapCY1 and ZapCY2 FRET reporters were stably produced in *S*. *pombe*, strains expressing ZapCY1 and ZapCY2 from the constitutive *pgk1* promoter were grown overnight in ZL-EMM, and the levels of each sensor examined by immunoblotting. Both reporters accumulated to similar levels in zinc-limited and zinc-replete cells indicating that their stability was not affected by zinc (Fig 2B). We also assessed the subcellular localization of each reporter in response to cellular zinc status using fluorescent microscopy. As shown in Fig 2C, there was strong fluorescence in cells expressing ZapCY1 or ZapCY2, which was absent from cells transformed with the empty vector. The ZapCY1 and ZapCY2 proteins were both localized to the cytosol and nucleus, and were excluded from the vacuole. For unknown reasons, higher levels of the ZapCY2 reporter accumulated in the nucleus of zinc-replete cells.

A potential concern with using ZapCY1 and ZapCY2 to assess alterations in the labile pools of zinc in yeast is that both sensors bind zinc ions, which in turn might reduce the levels of zinc that are normally available for cellular metabolism. In previous studies we have shown that the expression of the Loz1 target genes *zrt1* and *zym1* is dependent upon intracellular zinc levels [22]. Specifically, *zrt1* is expressed in zinc-limited cells and *zym1* is expressed in zinc-replete cells. We therefore predicted that *zrt1* and *zym1* expression would be altered if the ZapCY1/2 FRET sensors interfere with zinc homeostasis. As shown in Fig 2D, the introduction of the ZapCY1/2 FRET sensors had no major effect on *zym1* and *zrt1* mRNA levels. In addition, when the FRET reporters were co-expressed with a *zrt1-lacZ* reporter, there were no differences in β-galactosidase activity when compared to cells expressing the vector (Fig 2E). Taken together the above results show that the ZapCY1/2 FRET sensors accumulate within the cytosol and nucleus of cells without any substantial effect on zinc homeostasis.

**Fig 3:**
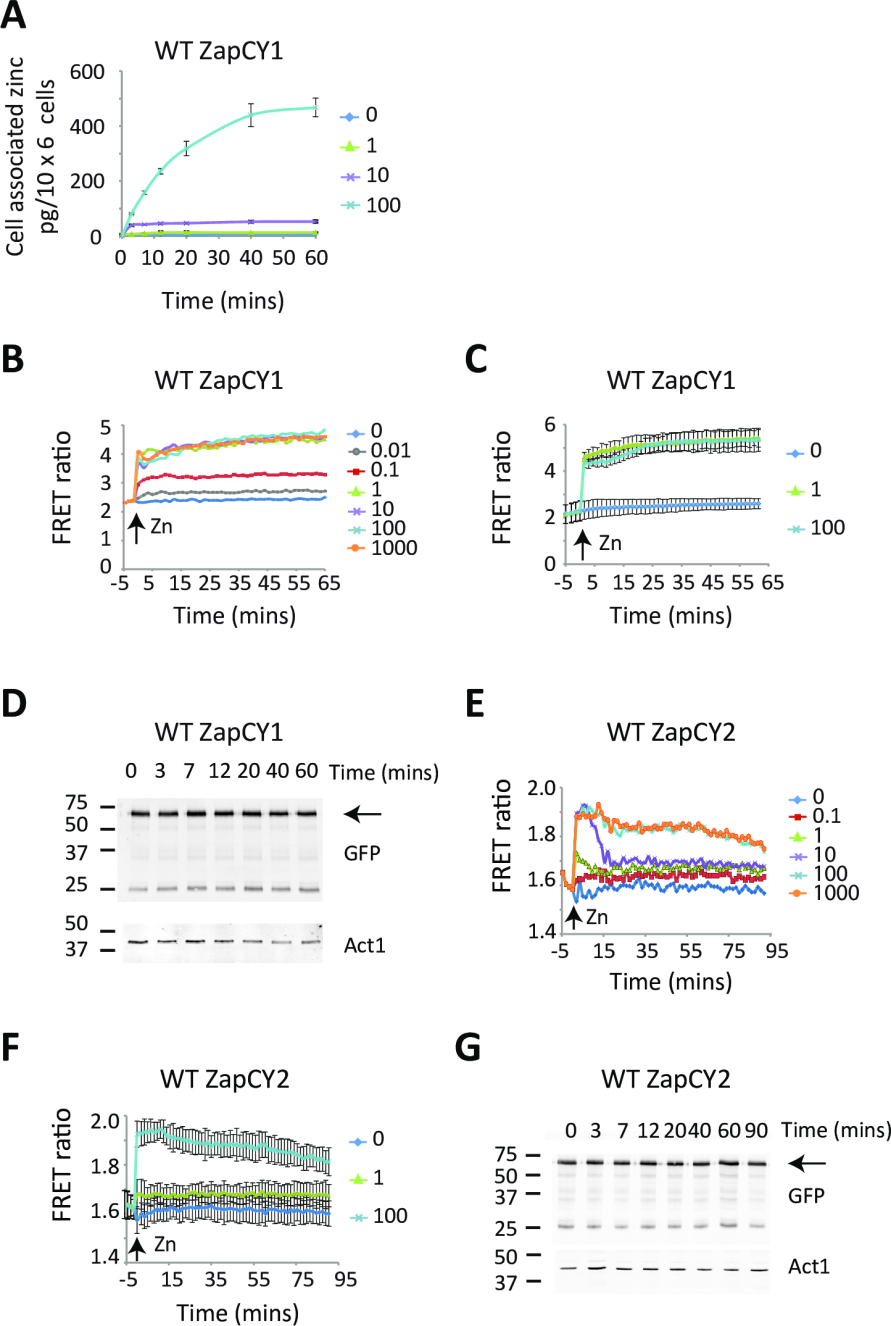
FRET responses of wild-type cells expressing ZapCY1 or ZapCY2 during zinc shock. **(A)** Wild-type cells expressing ZapCY1 were grown overnight in ZL-EMM. At t = 0 cells were exposed to 0, 1, 10, or 100 μM. Cells were harvested at the indicated time points and total cellular zinc measured using Atomic Absorption Spectroscopy. The final concentration of zinc/cell was calculated by comparing values to a standard curve. **(B and C)** Wild-type cells expressing ZapCY1 were grown overnight in ZL-EMM. Cells were transferred to temperature-controlled cuvettes and were assayed for FRET by spectrofluorometry. At t = 0 cells were shocked with 0–1000 μM Zn^2+^ and the changes in FRET monitored over time. The FRET ratio was determined by dividing the FRET emission at 535 nm by the eCFP emission at 475 nm following excitation of samples at 434 nm. Panel B shows a representative experiment and panel C shows the average values from 3 independent experiments with error bars representing standard deviations. **(D)** Wild-type cells expressing ZapCY1 were grown overnight in ZL-EMM. At t = 0 cells were shocked with 100 μM Zn^2+^. At the indicated time point cells were harvested and crude protein extracts prepped for immunoblot analysis. Immunoblots were incubated with antibodies to GFP and loading control Act1. **(E and F)** Wild-type cells expressing ZapCY2 were grown overnight in ZL-EMM. At t = 0 cells were shocked with 0–1000 μM Zn^2+^ and the FRET response measured as described in panel B. Panel E shows a representative experiment and panel F shows the average values from 3 independent experiments with error bars representing standard deviations. **(G)** Wild-type cells expressing ZapCY2 were grown overnight in ZL-EMM. At t = 0 cells were shocked with 10 μM Zn^2+^. Crude protein extracts were then prepped for immunoblot analysis as described in panel D.

To determine if the ZapCY1 and ZapCY2 FRET sensors are able to detect dynamic changes in the labile pool of zinc in fission yeast, we measured the activity of each reporter in vivo following a ‘zinc shock’. In a zinc shock experiment cells are initially depleted of zinc by growing overnight in ZL-EMM, which leads to increased expression of *zrt1* and high levels of Zrt1 on the plasma membrane. As zinc-limited cells are primed and ready to uptake zinc, zinc rapidly enters cells in a dose-dependent manner when it is added to the growth medium (Fig 3A). To examine the response of the high affinity FRET sensor to zinc shock, wild-type cells expressing ZapCY1 were grown overnight in ZL-EMM before being transferred to temperature-controlled microplate wells. Cells were excited at 434 nm and a FRET ratio calculated by dividing the intensity of the emission at 535 nm by the emission at 475 nm. The growth overnight in ZL-EMM resulted in a FRET ratio of 2.29 +/- 0.20 (Fig 3B and 3C, t = -5 min). This ratio remained constant until the addition of zinc, which led to a rapid increase in FRET in a dose-dependent manner (Fig 3B, 0.01–1 μM zinc). These changes in FRET occurred without affecting the stability of ZapCY1 (Fig 3D), consistent with the ZapCY1 sensor binding zinc and forming the closed conformation which brings YFP and CFP closer together. Zinc shocks with higher levels of zinc (10–1000 μM Zn^2+^) also led to a rapid increase in the FRET ratio (Fig 3B and 3C). However, the magnitude of the response was similar to that seen following a zinc shock with 1 μM zinc. We conclude from these results that a zinc shock with 1 μM Zn^2+^ results in sufficient levels of zinc accumulating within the cytosol and nucleus of cells to saturate the high affinity ZapCY1 sensor.

To assess the effects of a zinc shock on the low affinity reporter, similar experiments were performed with wild-type cells expressing ZapCY2. In these cells, growth overnight in ZL-EMM resulted in an initial FRET ratio of 1.57 +/- 0.05 (Fig 3E and 3F, t = 0 min). As ZapCY2 binds zinc with a lower affinity than ZapCY1, we predicted that higher levels of zinc would be needed to saturate ZapCY2. Consistent with this hypothesis, no significant increase in FRET was observed following a zinc shock with 0.1 μM Zn^2+^ and a modest response was seen with 1 μM Zn^2+^. A rapid increase in the FRET ratio to 1.92 +/- 0.03 was detected following a zinc shock with 10 μM Zn^2+^ (Fig 3E, t = 2 min). However, this FRET signal subsequently decreased until it reached a more constant ratio of ~ 1.75 after 15 minutes (Fig 3E, t = 15–90 min). As the zinc shock did not affect the stability of ZapCY2 (Fig 3G), these results are consistent with ZapCY2 rapidly binding zinc, and then zinc being lost to higher affinity zinc binding sites in the surrounding environment. Zinc shocks with higher levels of zinc (Fig 3E and 3F, 100 μM zinc) were sufficient to saturate the FRET sensor, but in contrast to ZapCY1, the FRET ratio slowly decreased with time. Thus, the ZapCY1 and ZapCY2 sensors are both able to detect changes in cytosolic zinc levels. However, higher levels of zinc are necessary to saturate ZapCY2 and the zinc bound to ZapCY2 is more readily lost to the surrounding environment.

### The FRET signal in cells expressing ZapCY1 and ZapCY2 is dependent upon *zrt1* expression

**Fig 4:**
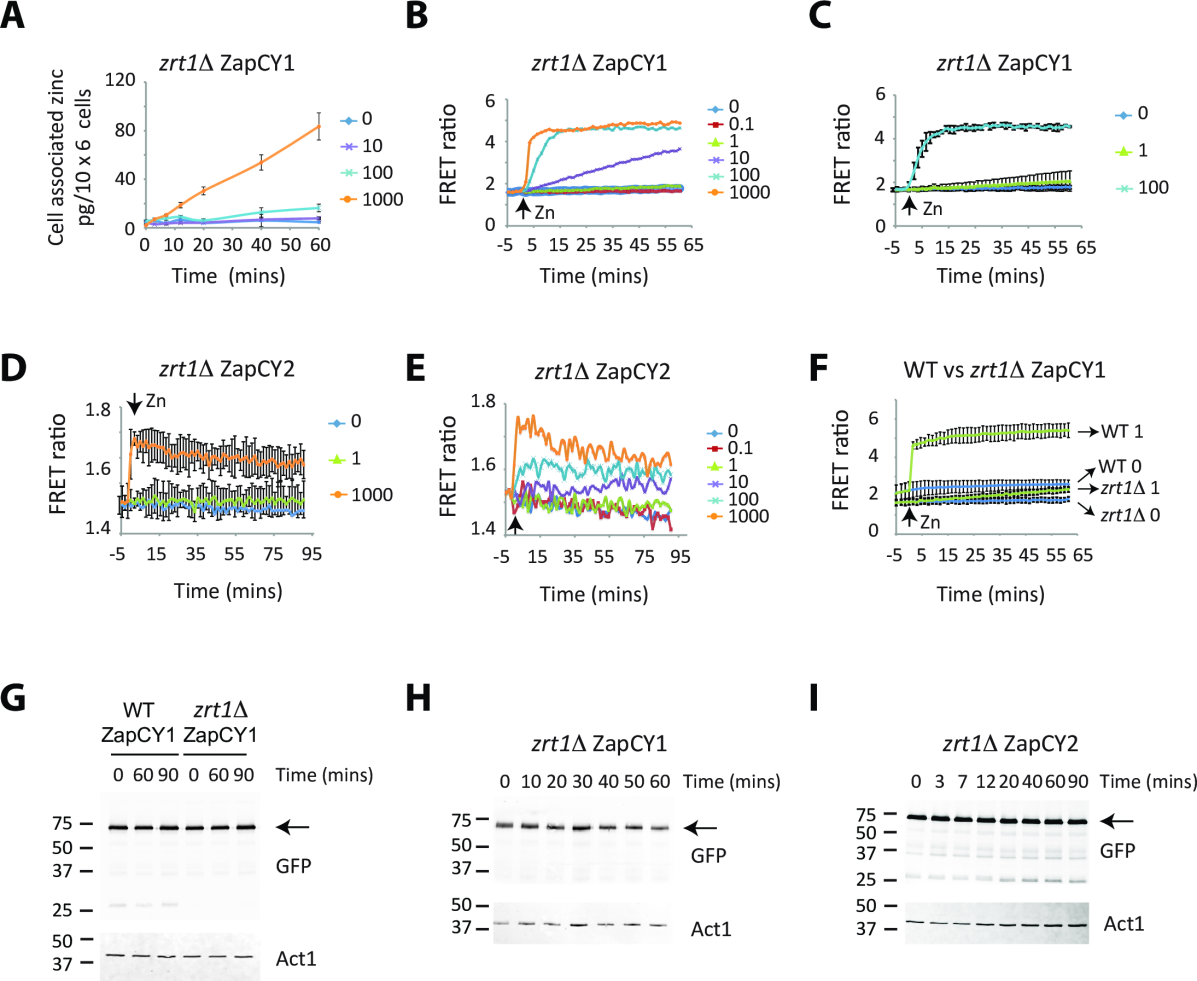
*zrt1*Δ cells accumulate lower levels of zinc in the cytosol. **(A)** *zrt1*Δ cells expressing ZapCY1 were grown overnight in ZL-EMM. At t = 0 cells were exposed to 0–1000 μM. Total cellular zinc was then determined by AAS as described for Fig 3A. **(B-F)** *zrt1*Δ cells expressing ZapCY1 or ZapCY2 were grown overnight in ZL-EMM. Cells were transferred to temperature-controlled cuvettes and at t = 0 shocked with 0–1000 μM Zn^2+^. Changes in the FRET ratio were measured as described in Fig 3B. Panels B and D show representative experiments and panels C and E show the average values from 3 independent experiments with error bars representing standard deviations. Panel F shows a comparison of a 1 μM zinc shock in wild-type and *zrt1*Δ cells expressing ZapCY1 **(G-I)** Wild-type and *zrt1*Δ cells expressing ZapCY1 or ZapCY2 were grown overnight in ZL-EMM. At t = 0 cells were shocked with 100 μM Zn^2+^. Cells were harvested at the indicated time points and crude protein extracts prepped for immunoblot analysis. Immunoblots were incubated with antibodies to GFP and loading control Act1.

To gain further evidence that the sensors were measuring changes in cytosolic zinc levels, ZapCY1 and ZapCY2 were introduced into cells lacking *zrt1*. In the absence of Zrt1, higher levels of zinc are needed during a zinc shock experiment to see an increase in total cellular zinc because cells rely on low affinity systems for zinc uptake (compare Fig 3A to Fig 4A). We therefore predicted that higher levels of zinc would be necessary to saturate ZapCY1 and ZapCY2 in *zrt1*Δ. Consistent with this prediction, higher levels of zinc were required to obtain a maximal FRET ratio change in *zrt1*Δ cells compared to the wild-type (Fig 4B–4E). As an example, a zinc shock with 1 μM zinc was sufficient to saturate ZapCY1 in the wild-type, but did not affect the activity of the ZapCY1 reporter in *zrt1*Δ (Fig 4F). The differences in FRET response were a result of loss of *zrt1*, as both reporters were expressed at similar levels to the wild-type, and a zinc shock had no effect on the stability of either reporter (Fig 4G–4I). Together, these results indicate that the FRET signal in cells is dependent upon the expression of *zrt1* and also is consistent with the activity of both reporters being directly regulated by cytosolic zinc levels.

**Fig 5:**
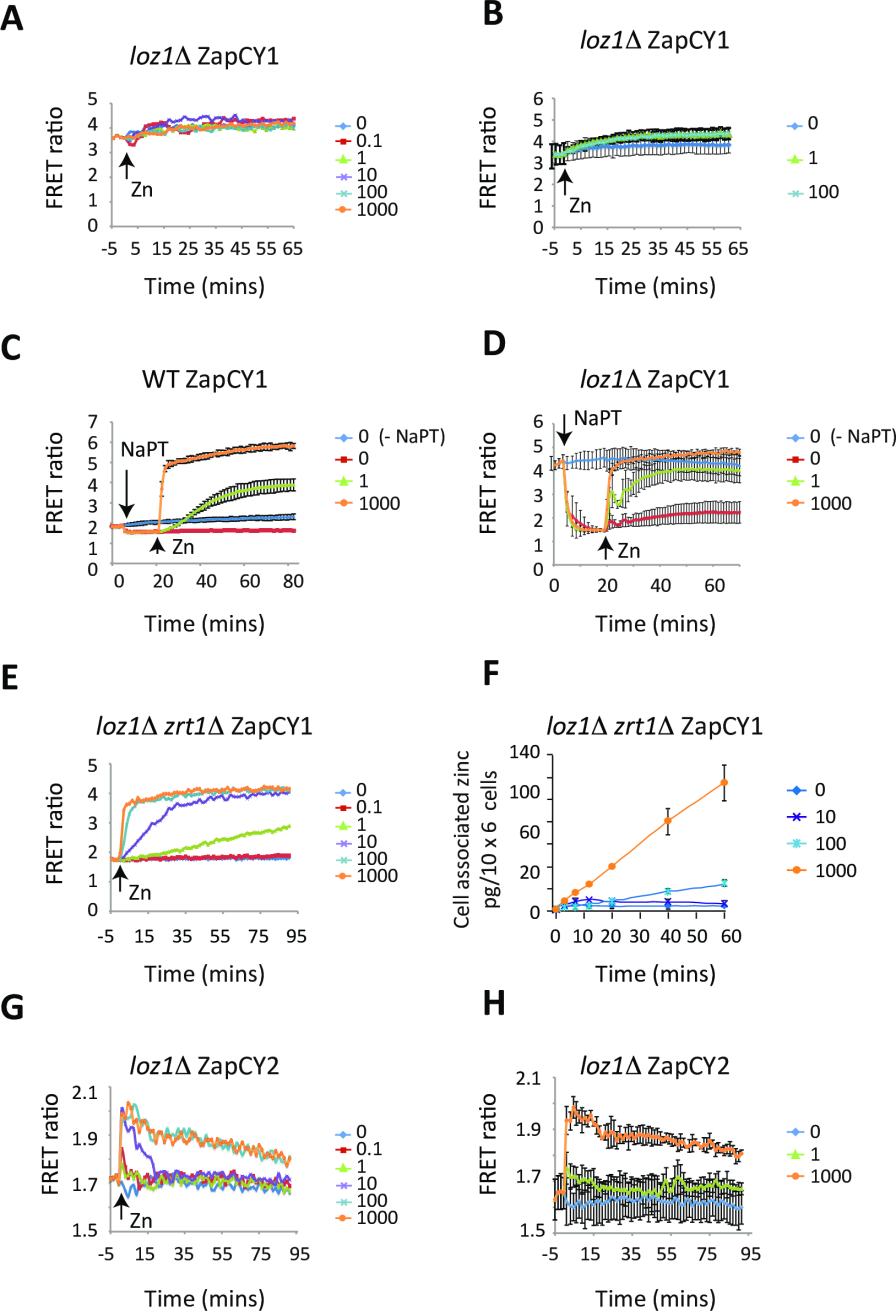
*loz1*Δ cells accumulate higher levels of zinc in the cytosol under zinc-limiting conditions. **(A and B**) *loz1*Δ cells expressing ZapCY1 were grown overnight in ZL-EMM and cells were transferred to temperature-controlled cuvettes. At t = 0 cells were shocked with 0–1000 μM Zn^2+^ and changes in the FRET ratio measured as described in Fig 3B. Panel A shows a representative experiment and panel B shows the average values from 3 independent experiments with error bars representing standard deviations. **(C and D)** Wild-type and *loz1*Δ cells expressing ZapCY1 were grown overnight in ZL-EMM. Cells were transferred to temperature-controlled cuvettes and at the indicated times were exposed to +/- 50 μM NaPT or 0, 1, or 1000 μM Zn^2+^. Changes in the FRET ratio were determined as described in Fig 3B. Results show the average values from 3 independent experiments with error bars representing standard deviations. **(E)** *loz1*Δ *zrt1*Δ cells expressing ZapCY1 were grown and subject to zinc shock as described in Fig 3B. A representative experiment is shown. **(F)** *loz1*Δ *zrt1*Δ cells were grown overnight in ZL-EMM. At t = 0 cells were exposed to 0–1000 μM. Total cellular zinc was then determined by AAS as described for Fig 3A. **(G and H)** *loz1*Δ cells expressing ZapCY2 were grown overnight in ZL-EMM. Cells were shocked with 0–1000 μM Zn^2+^ and changes in the FRET ratio measured as described in Fig 3B. Panel G shows a representative experiment and panel H shows the average values from 3 independent experiments with error bars representing standard deviations.

In our previous studies we found that genetic mutations that disrupt Loz1 function result in the constitutive de-repression of *zrt1* transcription, leading to increased expression levels. Therefore, to assess the effects of overexpression of *zrt1* on cytosolic zinc levels, ZapCY1 and ZapCY2 were introduced in *loz1*Δ cells and the FRET response was measured during a zinc shock experiment. Following the growth of *loz1*Δ ZapCY1 cells overnight in ZL-EMM, an initial FRET ratio of 3.3 +/- 0.5 was detected (Fig 5A and 5B), which is higher than the initial FRET ratio in cells expressing Loz1. Further, only a minor increase in FRET was seen following a zinc shock with 0.1–1000 μM zinc. As *loz1*Δ cells constitutively express *zrt1*, one explanation for the high initial FRET ratio in this mutant is that they have higher levels of zinc uptake leading to the saturation of ZapCY1. It was also possible that the ZapCY1 reporter was unable to respond to zinc in this genetic background. To distinguish between these possibilities, we used sodium pyrithione (NaPT) to artificially lower cytosolic zinc levels. Pyrithione is a membrane permeable ionophore that readily forms complex with zinc [23]. When 50 μM NaPT was added to wild-type ZapCY1 cells grown overnight in ZL-EMM, a small decrease in the FRET ratio consistent with this molecule binding or releasing accessible zinc within the cytosol (Fig 5C). Importantly, a rapid increase in FRET was seen when zinc was added to the NaPT treated cells. When a similar experiment was performed with *loz1*Δ cells, a large decrease in the FRET ratio was seen upon the addition of NaPT, which could be reversed by the addition of zinc (Fig 5D). These results indicate that the ZapCY1 reporter is functional in *loz1*Δ cells, and suggest that in the absence of strong chelators and ionophores it is saturated with zinc under all conditions.

To test whether the saturation of the ZapCY1 reporter in *loz1*Δ cells was a result of high *zrt1* expression, we examined the activity of the ZapCY1 reporter in double mutants lacking *loz1* and *zrt1*. In these cells a low FRET ratio of 1.8 +/- 0.2 was detected following growth overnight in ZL-EMM (Fig 5E). These results suggest that the high expression of *zrt1* significantly contributes to the saturation of ZapCY1 in *loz1*Δ cells. We also noted that higher levels of total zinc accumulated in *loz1*Δ *zrt1*Δ when compared to *zrt1*Δ following a zinc shock (Fig 5F). These results suggest that Loz1 controls the expression of a second lower affinity zinc uptake system and/or regulates the expression of other genes that affect cytosolic zinc availability. Consistent with this hypothesis, a zinc shock with 1 μM zinc did not result in an increased FRET ratio in *zrt1*Δ, but did lead to a slow increase in FRET in *loz1*Δ *zrt1*Δ (compare Figs 4B and 5E). Similarly, the ZapCY1 reporter was close to saturation after a 30 min zinc shock with 10 μM Zn in *loz1*Δ *zrt1*Δ cells, and yet in *zrt1*Δ, a zinc shock with 10 μM Zn zinc only led to a slow gradual increase in FRET over 60 min.

As *loz1*Δ cells accumulate higher levels of zinc within the cytosol, we also assessed the effects of this allele on the response of the low affinity ZapCY2 reporter. In contrast to the response of ZapCY1, the basal FRET ratio in zinc-limited *loz1*Δ ZapCY2 cells was similar to the wild-type (compare Figs 3F to 5H). The responses of the ZapCY2 reporter to zinc also resembled those of the wild-type (Fig 5G and 5H). Thus, under conditions of zinc deficiency, *loz1*Δ cells accumulate higher levels of zinc in the cytosol/nucleus relative to the wild-type. However, when zinc is not limiting in these cells it is effectively buffered and/or transported out of the cytosol.

### Cis4 and Zrg17 play a central role in the transport of zinc out of the cytosol during zinc deficiency

**Fig 6:**
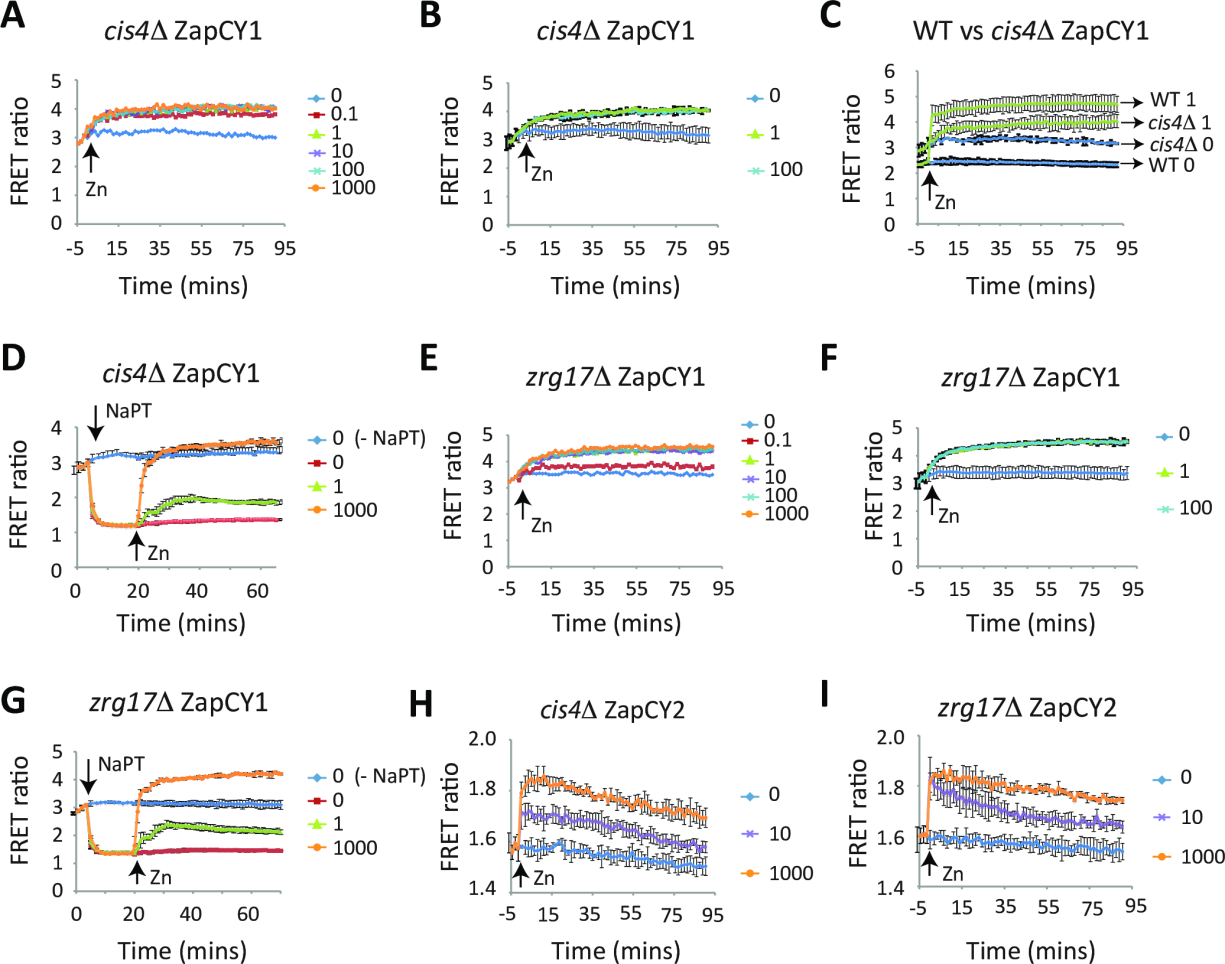
*cis4*Δ and *zrg17*Δ cells accumulate higher levels of zinc in the cytosol under zinc-limiting conditions. **(A, B, and C)** *cis4*Δ cells expressing ZapCY1 were grown and subject to zinc shock as described in Fig 3B. Panel A shows a representative experiment and panel B shows the average values from 3 independent experiments with error bars representing standard deviations. Panel C shows a comparison of a 1 μM zinc shock in wild-type and *cis4*Δ cells expressing ZapCY1. (D) *cis4*Δ ZapCY1cells were grown as described in Fig 3B. Cells were transferred to temperature-controlled cuvettes and at the indicated times were exposed to +/- 50 μM NaPT or 0, 1, or 1000 μM Zn^2+^. Changes in the FRET ratio were determined as described in Fig 3B. Results show the average values from 3 independent experiments with error bars representing standard deviations. **(E-G)** The FRET ratio was measured in *zrg17*Δ cells expressing ZapCY1 as described in panel C. **(H and I)** *cis4*Δ and *zrg17*Δ cells expressing ZapCY2 were grown and subject to zinc shock as described in Fig 3B. Each panel shows the average values from 3 independent experiments with error bars representing standard deviations.

The above results show that the ZapCY1 and ZapCY2 sensors can be used in *S*. *pombe* to measure dynamic changes in the levels of labile zinc in the cytosol and nucleus. As deletion of *cis4* or *zrg17* resulted in a growth defect on low zinc medium, we used these sensors to test whether Cis4 and Zrg17 were necessary for zinc transport out of the cell under this condition. When a zinc shock experiment was performed with *cis4*Δ ZapCY1 cells, the starting FRET ratio was higher than that observed in the wild-type. Additionally, only a small increase in the FRET ratio was seen with 0.1 μM zinc (from 2.8 +/- 0.23 to 4.0 +/-0.2) and also when cells were shocked with higher levels of zinc (1–1000 μM) (Fig 6A–6C). In contrast, the addition of NaPT resulted in a large decrease in the FRET ratio, which could be reversed by the addition of zinc (Fig 6D). For the most part, similar trends were seen with *zrg17*Δ ZapCY1 and *cis4*Δ *zrg17*Δ ZapCY1. However, for *zrg17*Δ cells the maximum FRET ratio was slightly higher than that observed with *cis4*Δ cells; and a zinc shock with 0.1 μM zinc was not sufficient to totally saturate ZapCY1 (Fig 6E–6G and S1 Fig). As ZapCY1 was largely saturated in *cis4*Δ and *zrg17*Δ cells following growth overnight in ZL-EMM, these results are consistent with Cis4 and Zrg17 being required for the transport of zinc out of the cytosol under conditions of zinc deficiency.

To determine if the higher levels of zinc that accumulated in *cis4*Δ and *zrg17*Δ also affected zinc binding to low affinity sites, similar experiments were performed with cells expressing ZapCY2. A zinc shock with 10 or 1000 μM zinc resulted in smaller increase in FRET compared to the wild-type (Fig 6H and 6I and S1 Fig). The signal also decreased over time. While it is possible that Cis4 and Zrg17 transport zinc out of the cytosol following a zinc shock, the decrease in FRET response in these mutants suggests that other mechanisms that are independent of Cis4 and Zrg17 protect the cytosol from accumulating high levels of labile zinc.

### Zhf1 transports labile zinc from the cytosol

**Fig 7:**
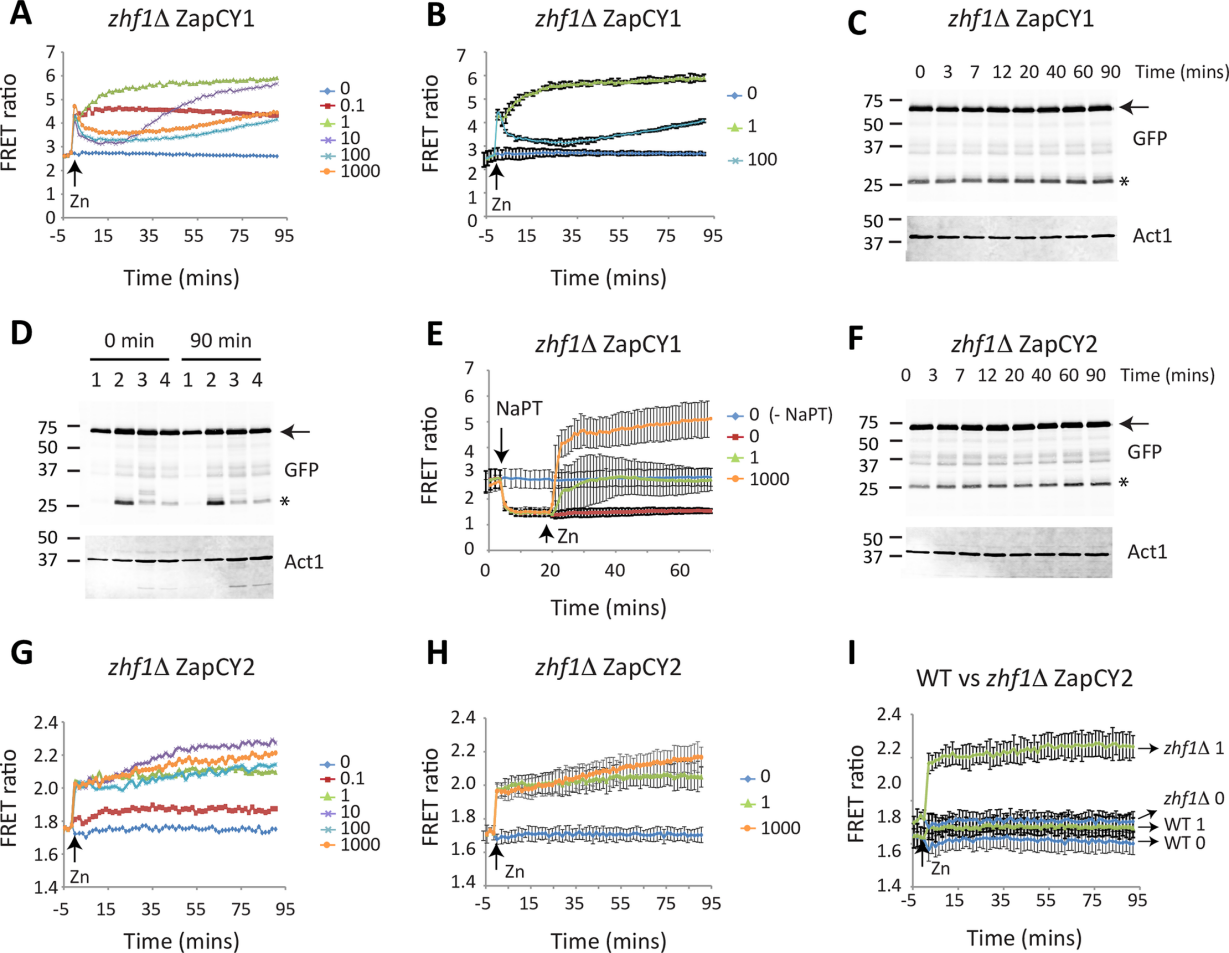
*zhf1*Δ cells accumulate higher levels of zinc in the cytosol following a zinc shock. **(A and B)** *zhf1*Δ cells expressing ZapCY1 were grown and subject to zinc shock as described in Fig 3B. Panel A shows a representative experiment and panel B shows the average values from 3 independent experiments with error bars representing standard deviations. **(C)** *zhf1*x expressing ZapCY1 were grown overnight in ZL-EMM. At t = 0 cells were shocked with 100μM Zn^2+^. Cells were harvested at the indicated time points and crude protein extracts prepped for immunoblot analysis. Immunoblots were incubated with antibodies to GFP and loading control Act1. **(D)** Wild-type (1), *zhf1*Δ (2), *cis4*Δ (3), and *zrg17*Δ (4) cells expressing ZapCY1 were grown overnight in ZL-EMM. Cells were harvested for immunoblot analysis following the overnight growth (t = 0) or after a zinc shock with 100 μM Zn^2+^ for 90 min (t = 90). Immunoblots were performed as described in panel C. **(E)** *zhf1*Δ ZapCY1cells were grown overnight in ZL-EMM. Cells were transferred to temperature-controlled cuvettes and were treated with +/- 50 μM NaPT for 20 min followed by the addition of 0–1000 μM Zn^2+^. Changes in the FRET ratio were determined as described in Fig 3B. **(F)** *zhf1*Δ cells expressing ZapCY2 were grown overnight in ZL-EMM. Cells were shocked with 100 μM Zn^2+^ and cell harvested for immunoblot analysis at the indicated time points. Immunoblots were performed as described in panel C. Results show the average values from 3 independent experiments with error bars representing standard deviations. **(G-I)** *zhf1*Δ cells expressing ZapCY2 were grown and subject to zinc shock as described in Fig 3B. Panel F shows a representative experiment and panel G shows the average values from 3 independent experiments with error bars representing standard deviations. Panel I shows a comparison of a 1 μM zinc shock in wild-type and *zhf1*Δ cells expressing ZapCY2.

As Zhf1 is predicted to play the primary role in protecting cells from zinc toxicity, we next examined the activity of ZapCY1 and ZapCY2 in strains lacking *zhf1*. In *zhf1*Δ ZapCY1 cells grown overnight in ZL-EMM, the basal FRET ratio and response of this sensor to zinc shocks with 0.1 and 1 μM zinc were similar to those seen in wild-type cells (Fig 7A and 7B). In contrast, a zinc shock with 10 μM zinc led to a rapid increase in the FRET ratio, followed by an immediate decrease. After these rapid changes the FRET ratio slowly increased for the remainder of the experiment. A similar response was seen with zinc shocks with higher levels of zinc, with the exception that it took longer (~30 min) to see the increase in FRET ratio. To test whether this atypical response was a result of the instability of the ZapCY1 reporter in *zhf1*Δ cells, immunoblot analysis was used to examine the stability of ZapCY1 during a zinc shock with 100 μM Zn^2+^. As shown in Fig 7C and 7D, elevated levels of a lower molecular weight band accumulated in this strain (see asterisk), suggesting that ZapCY1 was more prone to degradation in this strain relative to others. Despite this higher level of degradation, there were no changes in the levels of the full-length reporter and experiments using the zinc chelator NaPT resulted in FRET profiles that resembled those observed in the wild-type (Fig 7E). Although we do yet understand the zinc-dependent changes in the FRET response in *zhf1*Δ cells, the observation that they are not observed in the presence of NaPT suggests that they result from zinc accumulating in the cytosol of this strain.

To gain further evidence that Zhf1 protects the cytosol from excess zinc, similar experiments were performed with *zhf1*Δ ZapCY2. In these cells, zinc had no effect on the stability of the full length reporter and a zinc shock with 1 μM zinc was sufficient to saturate ZapCY2 (Fig 7F–7I). The FRET ratio after a zinc shock with 1 μM zinc also remained high for the duration of the experiment. These results reveal that higher levels of zinc accumulate in the cytosol of *zhf1*Δ following a zinc shock and indicate that Zhf1 has a central role in removing labile zinc from the cytosol.

### The expression of *cis4*, *zrg17*, and *zhf1* is not dependent upon zinc

**Fig 8:**
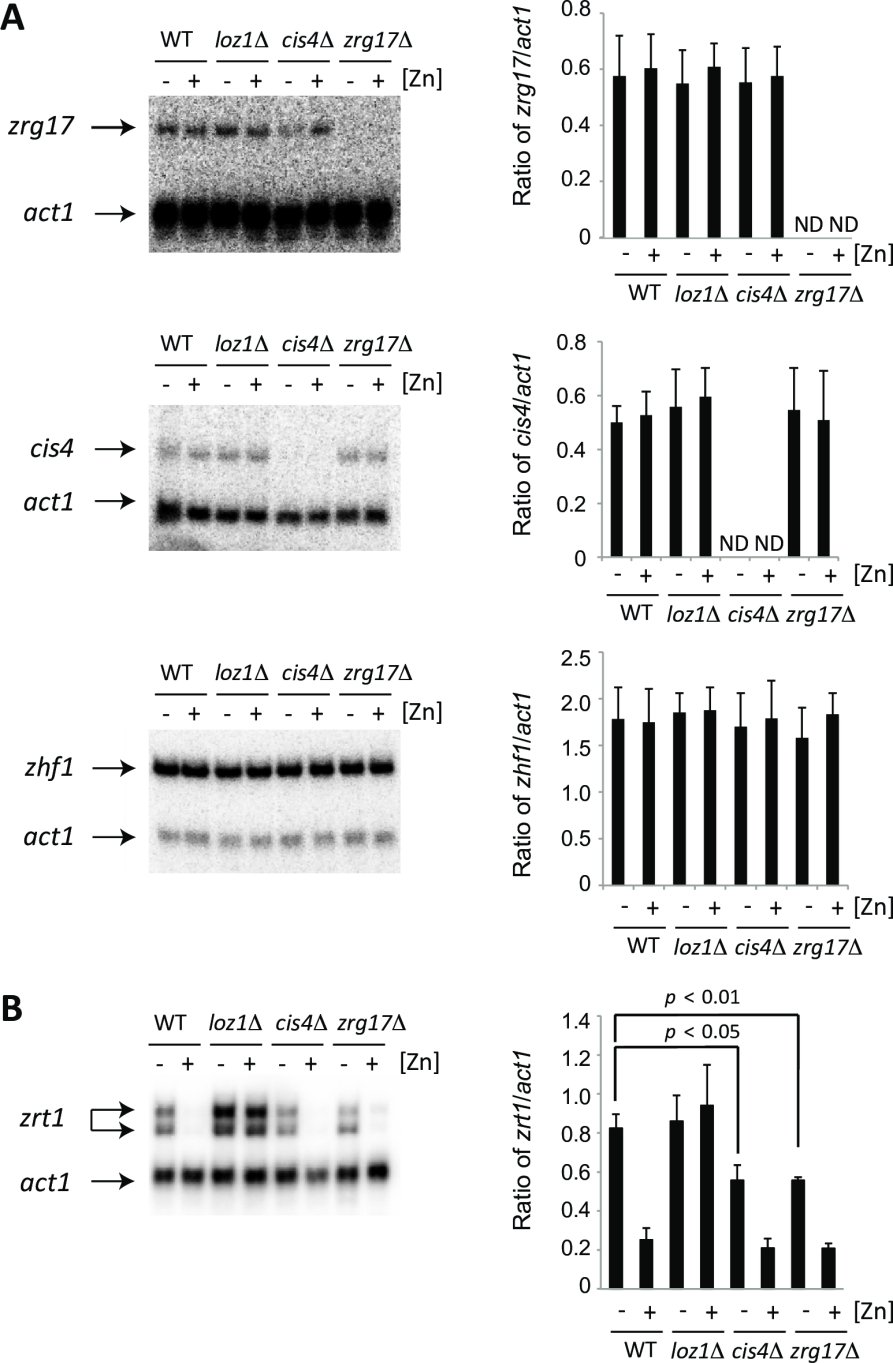
The expression of *cis4*, *zrg17*, and *zhf1* is not dependent upon zinc. **(A)** Total RNA was isolated from wild-type and the indicated mutants grown overnight in ZL-EMM supplemented with 0 or 100 μM Zn^2+^. The levels of *cis4*, *zrg17*, and *zhf1* transcripts were compared to the loading control *act1* using S1 nuclease analysis. A representative experiment is shown in the left panel and the average values from three independent experiments are shown on the right, with error bars representing standard deviations. ND = Not determined. (B). S1 nuclease analysis was performed as described above with the exception that mRNA was hybridized to probes complementary to *zrt1* and *act1*. Radiolabelled *act1* probes were diluted by 10-fold with unlabeled probe for experiments with *cis4*, *zrg17*, and *zhf1*. When cells were grown under zinc-limiting conditions, lower levels of *zrt1* transcript accumulated in the *cis4*Δ and *zrg17*Δ mutants compared to the wild-type. *p* values were determined using a student's t-test.

The above results suggest that the Cis4/Zrg17 complex plays a primary role in the transport of zinc out of a zinc-limited cytosol, while Zhf1 has the dominant role in transporting labile zinc from the cytosol. In *S*. *cerevisiae* the expression *ZRG17* increases under conditions of zinc deficiency and this increase is critical for normal endoplasmic reticulum function under this condition [11]. To determine if the expression of *cis4* and *zrg17* was dependent on zinc in fission yeast we used S1 nuclease analysis to examine mRNA levels in wild-type and *loz1*Δ cells grown under zinc-limiting and zinc-replete conditions. As shown in Fig 8A, *cis4* and *zrg17* transcripts accumulated under all conditions indicating that their expression is not affected by zinc or Loz1. The levels of *zhf1* mRNAs were also not regulated by zinc and Loz1, consistent with previous studies that have demonstrated experimentally that the expression of *zhf1* is not affected by cellular zinc status [13, 24].

As deletion of *cis4* or *zrg17* resulted in increased saturation of the high affinity ZapCY1 reporter, we also used S1 nuclease analysis to test whether these mutants accumulated sufficient levels of zinc in the cytosol to trigger increased Loz1-mediated gene repression. The rationale for these experiments is that Loz1 represses target gene expression when zinc levels are high. As a consequence, if higher levels of zinc accumulate in the cytosol of *cis4*Δ and *zrg17*Δ, this could result in increased repression of Loz1 target genes. When cells were grown under zinc-limiting conditions, lower levels of *zrt1* transcripts accumulated in *cis4*Δ and *zrg17*Δ cells relative to the wild-type control (Fig 8B). These results are consistent with the Cis4/Zrg17 complex transporting zinc out of cytosol of zinc-limited cells.

## Discussion

Yeast are useful model systems to study zinc homeostasis, as they are able to survive in low zinc environments and rapidly adapt to conditions of zinc excess. In this work we took advantage of these properties by examining the activity of the zinc-responsive ZapCY FRET reporters following overnight growth in a zinc-limited medium and during a zinc shock. We show that ZapCY1 and ZapCY2 are both able to measure dynamic changes in cytosolic zinc levels in fission yeast and that higher levels of zinc are necessary to saturate ZapCY2. We also show that there is a transient increase in FRET following a zinc shock in wild-type cells expressing ZapCY2, suggesting that zinc bound to this sensor exchanges with other ligands within the cytosol that can bind or buffer zinc. As ZapCY1 is able to detect zinc ions binding to high affinity sites within proteins, and ZapCY2 detects binding to low affinity sites, these sensors create useful tools for monitoring the factors that influence cytosolic zinc ion availability and zinc ion binding within the cytosol.

**Fig 9:**
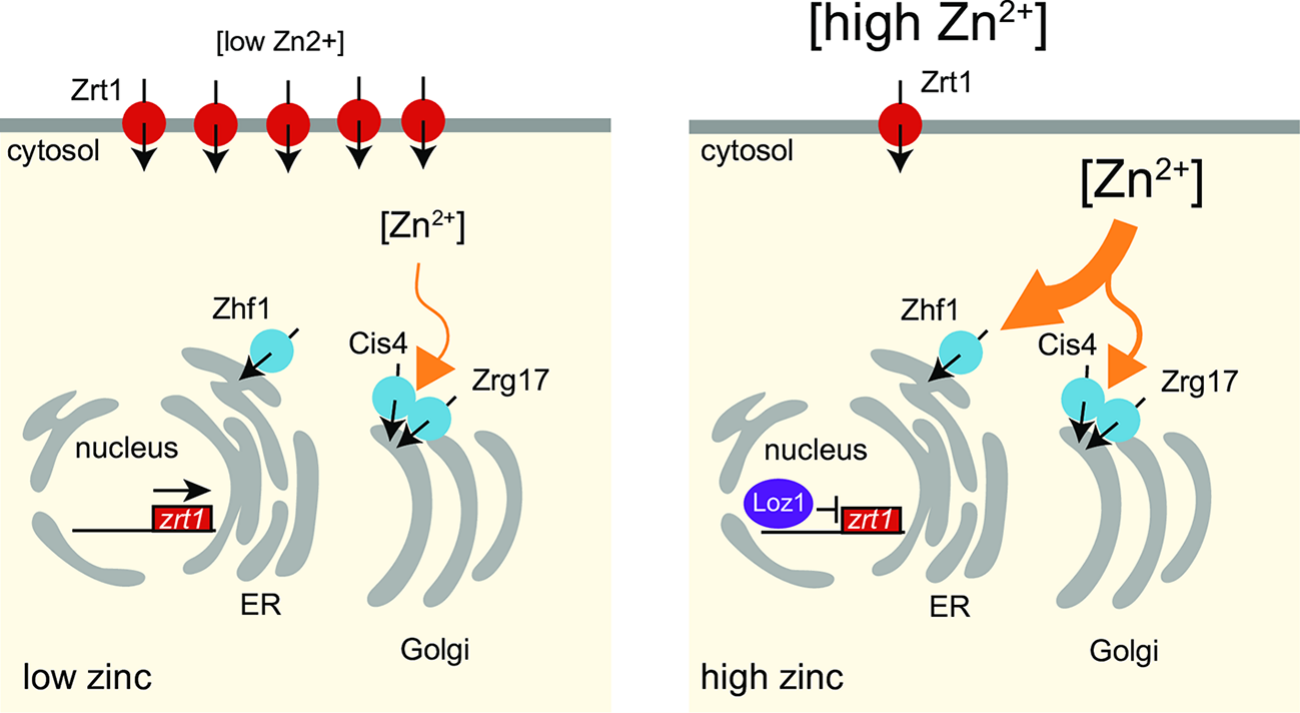
The Cis4/Zrg17 heterodimer and Zhf1 have complementary roles in transporting zinc out of the cytosol into the secretory pathway. When intracellular zinc levels are low, the Cis4/Zrg17 heterodimer transports zinc out of the cell into the secretory pathway (left panel). When zinc is not limiting, Zhf1 plays the primary role in transporting zinc out of the cytosol into cellular zinc stores (right panel). See text for further details.

To identify additional factors that affect the levels and availability of zinc within the cytosol, we used ZapCY1/2 to test whether Cis4, Zrg17, and Zhf1 have redundant or complementary roles in zinc transport out of the cytosol. We found that deletion of *cis4* or *zrg17* resulted in higher levels of saturation of ZapCY1 under conditions of zinc deficiency, whereas deletion of *zhf1* had little effect on the saturation of ZapCY1 under this condition. In contrast, significantly lower levels of zinc were necessary to saturate ZapCY2 in *zhf1*Δ cells compared to *cis4*Δ or *zrg17*Δ following a zinc shock. We propose that the Cis4/Zrg17 heterodimer preferentially transports zinc out of the cytosol into the secretory pathway under zinc-limiting conditions, whereas Zhf1 has the dominant role in transporting labile zinc out of the cytosol when zinc is not limiting (Fig 9). In this model, the transport activity of the Cis4/Zrg17 heterodimer ensures that zinc is supplied to the secretory pathway under zinc-limiting conditions. As a reduction in cytosolic zinc levels also triggers the inactivation of Loz1 and increased expression of the *zrt1* zinc uptake system, cells are able to balance the levels of zinc uptake with zinc flux out of the cytosol. Cells face a different challenge when zinc is in excess, as too much zinc is toxic to cell metabolism. Under these conditions, the dominant role of Zhf1 results in excess zinc being directed to intracellular stores, protecting the cytosol and other organelles from the toxic effects of too much zinc.

A key question that our studies raise is what is the mechanism by which individual CDF family members preferentially transport zinc under zinc-limiting or zinc-replete conditions? Studies with *S*. *cerevisiae* have revealed much of what we know about the ability of CDF proteins to transport zinc under varying conditions of zinc stress. In this yeast, the Msc2/Zrg17 complex facilitates the transport of zinc into the endoplasmic reticulum, whereas Zrc1 and Cot transport zinc into the vacuole [10, 14]. One factor that affects zinc transport via the Msc2/Zrg17 complex is *ZRG17* expression. *ZRG17* is a Zap1 target gene that is expressed at higher levels in zinc-deficient cells [7, 11]. Importantly, in the absence of the Zap1-dependent induction of *ZRG17*, zinc-deficient cells experience greater levels of ER stress [11]. These results suggest that the levels of Zrg17 protein limit zinc transport by the Msc2/Zrg17 complex and that the increase in *ZRG17* expression is critical for normal ER function under conditions of zinc-deficiency. Recent studies have also revealed that higher levels of *MSC2* mRNAs accumulate in zinc-deficient cells [25]. The levels of Msc2 may also be an important factor that limits zinc transport by the Msc2/Zrg17 complex. While changes in gene expression are an integral part of zinc transport into the ER under zinc-deficient conditions, it is also important to note that *ZRC1* is a Zap1 target gene, and yet overexpression of *ZRC1* does not affect cytosolic zinc availability in zinc-deficient cells [26]. These results suggest that Zrc1 does not play a significant role in transporting zinc out of the cytosol under this condition. They also reveal that increased expression of a zinc transport gene does not necessarily result in more zinc being transported out of the cytosol, and that other factors likely affect zinc transporter function.

As the expression of *cis4*, *zrg17*, and *zhf1* is not dependent upon zinc, what other factors could affect their ability to transport zinc out of the cytosol? One possibility is that there are intrinsic differences in the ability of Zhf1 and Cis4/Zrg17 to transport zinc. For example, if the affinities of the zinc binding sites on the cytosolic face of Zhf1 were weaker than those of the Cis4/Zrg17 heterodimer, this latter complex may only be able to acquire zinc when the cytosolic zinc pools are less saturated. An alternative possibility is that the labile pool of zinc that is accessible to Zhf1 under zinc-limiting conditions is dependent on the presence of an active Cis4/Zrg17 heterodimer. Potential mechanisms that could lead to a pool of labile zinc that is inaccessible to Zhf1 include an increase in the total number of buffering components in the cytosol (i.e. the total amount of zinc remains the same and the buffering capacity increases) and/or tighter buffering of cytosolic zinc (preventing zinc from being available to the weaker binding sites of Zhf1). While future experiments are necessary to determine the precise nature of the buffering components of the cytosol, and the affinity of different CDF transporters for zinc, it is noteworthy that Cis4/Zrg17 and Msc2/Zrg17 complexes both appear to have a primary role in transporting zinc out of the cytosol into the secretory pathway under zinc-limiting conditions. In addition, Zhf1 and Zrc1 both facilitate the transport of zinc from the cytosol when zinc availability is not limited. These similar functions suggest that at least some features of these transporters are conserved in fission and budding yeast.

In addition to the potential differences above, multiple other factors could affect the function of the Cis4/Zrg17 heterodimer and Zhf1. For example, in yeast and humans, zinc transporters from the ZIP family are targeted for degradation in response to high zinc [27, 28]. Although it is currently not known if the stability or activity of each of the *S*. *pombe* CDF proteins are regulated at a post-translational level in response to cellular zinc levels, proteomic studies that compared the copy numbers of proteins in fission yeast during vegetative growth in minimal medium revealed the presence of ~12,000–14,000 Zhf1 molecules/cell, ~3000–6000 Zrg17 molecules/cell, and ~ 1300–5500 Cis4 molecules/cell [29, 30]. The higher levels of Zhf1 relative to Zrg17 and Cis4 may therefore be one factor that contributes to Zhf1 having the principal role in transporting zinc out of the cytosol in a zinc-replete environment. In addition to factors that directly affect the function of zinc transport proteins, it is unclear if the subcellular localization of zinc transporters or their local environment affects their function. Moreover, relatively little is known about the molecules that buffer zinc within organelles and the cytosol, and whether the buffering capacity of an organelle for zinc, and/or the number and affinity of zinc-binding proteins within a compartment influences zinc transport. Thus, future studies with CDF proteins from *S*. *pombe* and other organisms are warranted to identify additional factors that alter zinc transport function.

We also examined the effects of the *loz1*Δ allele on cytosol zinc availability. We had previously found that *loz1*Δ cells constitutively express *zrt1* and hyperaccumulate zinc when excess zinc is present in the growth medium [22]. Although *loz1*Δ cells hyperaccumulate zinc, paradoxically they have a more severe growth defect under zinc-deficient conditions compared to zinc replete (Fig 1). Here we find that the *loz1*Δ allele results in the saturation of the high affinity ZapCY1 sensor following growth overnight in ZL-EMM, indicating that this mutant accumulates higher levels of zinc in the cytosol relative to the wild-type. We also find that the response of the ZapCY2 reporter was similar to that of the wild-type, revealing that labile zinc entering *loz1*Δ cells is rapidly removed into stores and/or is effectively buffered. These latter results reveal that other mechanisms that are independent of Loz1 help *S*. *pombe* to maintain zinc homeostasis. They also provide an explanation for the viability of the *loz1* mutant in high zinc. Another observation that we made was that ZapCY1 was not saturated in double mutants lacking *zrt1* and *loz1*, and that this double mutant accumulated higher levels of zinc in the cytosol relative to *zrt1*. These results indicate that the constitutive derepression of *zrt1* is the primary reason for the saturation of ZapCY1 in *loz1*Δ cells. They also suggest that Loz1 regulates other genes that affect cytosolic zinc availability. Known Loz1 target genes include *zrt1*, as well as *adh4* (alcohol dehydrogenase 4), *gcd1* (glucose dehydrogenase 1), and *SPBC1348*.*06c*, which encodes a small fungal protein of unknown function [22, 31]. Loz1 also represses the expression of non-protein coding RNAs that interfere with the expression of the *adh1* (alcohol dehydrogenase 1) and *zym1* (zinc metallothionein 1) genes [22, 32]. The expression of *adh1* and *zym1* is therefore inverse to that of other Loz1 targets, in that they are repressed under conditions of zinc deficiency. Although no known Loz1 target gene other than *zrt1* has a role in transporting zinc, altered expression of some of its targets could affect intracellular zinc availability. For example, as the *loz1*Δ allele results in the constitutive repression of *adh1*, which encodes the abundant zinc binding protein Adh1, the lower levels of this protein could result in higher levels of zinc being available for other proteins. Thus, future studies to identify new Loz1 target genes and to examine the roles of existing target genes in controlling intracellular zinc availability may provide additional insight into factors affecting zinc homeostasis.

The ability of some CDF proteins to transport zinc is a manner that is dependent upon the levels of ‘labile’ or ‘readily available’ zinc in the cytosol could be of particular importance in organisms that express large numbers of CDF family members. For example, humans express 10 CDF family members (named ZnT1-10), while *Arabidopsis thaliana* and *C*. *elegans* each express 14 family members [33]. Potential reasons for why these organisms have so many CDF proteins include that they have unique subcellular localizations, different metal ion specificities, and/or that they have more specialized roles in supplying zinc to smaller subsets of proteins [5, 34–38]. Some genes encoding CDF proteins also show tissue- or developmental- specific expression patterns, while others are regulated by zinc and/or by hormonal or stress-responsive signaling pathways [5, 39, 40]. Although it is currently unclear in other organisms if the activity of specific CDF proteins is dependent upon cellular zinc status, the conserved role of this family in supplying zinc to organelles and storage compartments raises the possibility that the activity of other CDF proteins may also be fined tuned according to cytosolic zinc ion availability.

## Materials and methods

### Yeast strains and growth conditions

To generate the strains used for the FRET analysis, the plasmids pZapCY1 and pZapCY2 were linearized with NruI and were integrated into the *leu1-32* locus of the wild-type strain JW81 (*h- ade6-M210 leu1-32 ura4-D18*) [41]. All other strains expressing ZapCY1 or ZapCY2 were generated from genetic crosses with the wild-type ZapCY1 (ABY795) or WT ZapCY2 (ABY797) with the respective mutant. The strains co-expressing the ZapCY1 and ZapCY2 with the *zrt1-lacZ* reporter were generated from genetic crosses with JW81 containing the integrated reporter TN-*zrt1-lacZ* [24]. To generate zinc-deficient and zinc-replete cells, yeast strains were initially grown to exponential phase in the nutrient rich YES medium. Cells were then spun down and washed twice in ZL-EMM, a derivative of Edinburgh minimal medium that lacks zinc (ZL-EMM). Washed cells were then diluted to 0.02 OD_600_ with fresh ZL-EMM and were grown for 16 hrs at 31°C in ZL-EMM or in this medium supplemented with 1, 10, or 100 μM ZnCl_2_. For all zinc shock experiments, cells were grown as described above in ZL-EMM without zinc. The indicated amount of zinc (0.01–1000 μM ZnCl_2_) was then added to induce the zinc shock.

### Plasmid construction

The plasmids pZapCY1 and pZapCY2 were generated by PCR amplifying the coding regions for ZapCY1 and ZapCY2 from the vectors pcDNA3.1-ZapCY1 and pcDNA3.1-ZapCY2 respectively, with primers containing EcoRI and BamHI restriction sites. The ZapCY1/2 PCR products were then digested with EcoRI and BamHI and cloned into similar sites into the vector JK-pgk1-adh4T. The vector JK-pgk1-adh4T is a derivative of JK148 that contains 840 bp of the *pgk1* promoter inclusive of its 5’UTR and 726 bp of the *adh4* terminator. It was generated by initially PCR amplifying the *pgk1* promoter with primers containing KpnI and EcoRI restriction sites. KpnI- and EcoRI- digested PCR products were then cloned into the vector JK148 to generate JK-pgk1. The *adh4* terminator was cloned using a similar approach with the exception that primers were designed to introduce the *adh4* PCR product into the BamHI/SacI sites of JK-pgk1.

### β-Galactosidase assays and Atomic Absorption Spectroscopy (AAS)

**β**-Galactosidase assays were performed as described previously [42]. Activity units were calculated as follows: (ΔA420 x 1000)/(min x ml of culture x culture absorbance at 600 nm). For AAS 10 ml of cells were grown in ZL-EMM as described above. After the OD600 was measured, the indicate amount of zinc was added at t = 0 min. Cells were then grown at 31°C with shaking and 1.5 ml aliquots removed at the indicated time point. To remove extracellular zinc, cells were washed twice with 0.5 M EDTA and twice with ddH_2_O. Cell pellets were then digested by boiling in 150 μl of metal free nitric acid for 45 min and the zinc content measured using a SpectrAA 220FS Atomic Absorption Spectrometer. The final zinc concentration/cell was calculated by comparing the readings to a standard curve generated using a zinc standard (Sigma 18827). All values are the average from three independent experiments and error bars represent standard deviations.

### FRET Measurements

For FRET experiments, cells were grown for 16 hrs in ZL-EMM as described above. ~2.5 x 10^6^ cells were directly transferred to temperature controlled 96 well plates and the FRET emission intensities measured using spectrofluorometry using the following excitation and emission wavelengths: eCFP excitation 434 nm / emission 474 nm, and FRET excitation 434 nm / emission 535 nm. The FRET ratio was calculated by dividing the FRET emission intensity by the eCFP emission intensity. All average values show the mean FRET ratio from three independent experiments that were performed on independent days. Error bars show standard deviations.

### Immunoblotting, RNA blotting, S1 nuclease assays and microscopy

For immunoblotting, total protein extracts were prepared by a trichloroacetic acid precipitation. Proteins were separated by SDS/PAGE analysis using a 10% resolving gel before transfer to nitrocellulose membranes. Proteins were detected using anti-GFP (Sigma G1544) or anti-Actin (Abcam ab3280), and secondary antibodies IR-Dye800CW conjugated anti-mouse IgG (LICOR) and IRDye680 conjugated anti-rabbit IgG (LICOR). Signal intensities were measured using an Odyssey infrared imaging system. For RNA analysis, total RNA was purified using hot acidic phenol method. RNA blots and S1 nuclease analyses were performed as described previously [32, 43]. Probes for the RNA blot analyses were generated using the MAXISCRIPT T7 kit (Ambion) according to manufacturers instructions, whereas probes for the S1 nuclease analyses were generated by 5’ end labeling the following oligonucleotides: *zrg17* 5’-GATCACTAATAGTTACAGAGACATTATTATTTATAGGGTTTTGAATCTGAATAGCAGTCGGGATG- 3’, *cis4* 5’- CGAACGCAGAAGAATTAACATTCATTTTTGTCGTCAGGAACACCCAAAAGCTGTGGTTGAC-3’, *zhf1* 5’-GTTGCCAGCCATATGTGTATTTTGGTTCGTGAGATGTTGAATGTGCTAGACGAGTAGCCCA-3’, *zrt1* 5’- CCATATTCGTTGAATTCATTGGCATCACCTCCACAAGTCACAGTAGCAGAGCTATCATCGTC-3’, and *act1* 5’-GTCCCATACCTACCATAATACCATGGTGACGGGGTCTACCGAC-3’. Act1 probes were diluted with unlabeled probe where indicated. Fluorescent microscopy of live cells was performed with an Olympus FV 1000 Filter Confocal system, using filter sets for GFP.

### Statistical analysis

Data are presented as the mean ± standard deviation (SD). Statistical analyses were performed using GraphPad Prism 5 software (GraphPad Software, La Jolla, CA, USA). Where appropriate, data were analyzed by a Student unpaired t-test. A p value of <0.05 was considered statistically significant.

## Supporting information

S1 Fig *cis4*Δ *zrg17*Δ double mutants accumulate higher levels of zinc in the cytosol under zinc-limiting conditions. *cis4*Δ *zrg17*Δ cells expressing ZapCY1 **(A)** or ZapCY2 **(B)** were grown overnight in ZL-EMM. Cells were transferred to temperature-controlled cuvettes and were assayed for FRET by spectrofluorometry. At t **=** 0 cells were shocked with the indicated amount of Zn^2+^ and the changes in FRET monitored over time. The FRET ratio was determined by dividing the FRET emission at 535 nm by the eCFP emission at 475 nm following excitation of samples at 434 nm. Results represent the average values from 3 independent experiments with error bars representing S.D.
(TIF)
