# Supplementary figures and images for: Zinc transporters belonging to the Cation Diffusion Facilitator (CDF) family have complementary roles in transporting zinc out of the cytosol
Source: PLoS Genet. 2018 Mar 12;14(3):e1007262. doi: 10.1371/journal.pgen.1007262 (PMC5864093; doi:10.1371/journal.pgen.1007262)

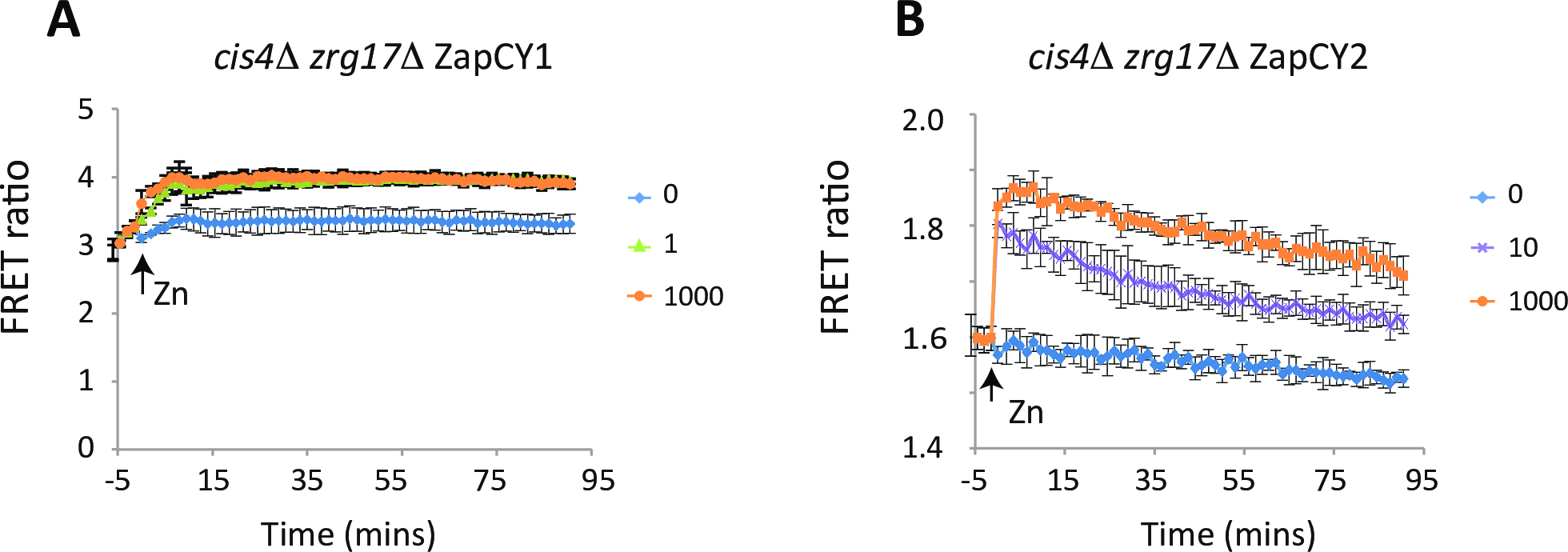

Supplement: S1 Fig — cis4Δ zrg17Δ cells expressing ZapCY1 (A) or ZapCY2 (B) were grown overnight in ZL-EMM. Cells were transferred to temperature-controlled cuvettes and were assayed for FRET by spectrofluorometry. At t = 0 cells were shocked with the indicated amount of Zn2+ and the changes in FRET monitored over time. The FRET ratio was determined by dividing the FRET emission at 535 nm by the eCFP emission at 475 nm following excitation of samples at 434 nm. Results represent the average values from 3 independent experiments with error bars representing S.D. (TIF) [file pgen.1007262.s001.tif]
